# y+LAT1 and y+LAT2 contribution to arginine uptake in different human cell models: Implications in the pathophysiology of Lysinuric Protein Intolerance

**DOI:** 10.1111/jcmm.14801

**Published:** 2019-11-09

**Authors:** Bianca Maria Rotoli, Amelia Barilli, Rossana Visigalli, Francesca Ferrari, Valeria Dall’Asta

**Affiliations:** ^1^ Laboratory of General Pathology Department of Medicine and Surgery (DiMec) University of Parma Parma Italy

**Keywords:** lysinuric protein intolerance, SLC7A6, SLC7A7, system B0,+, system y+L

## Abstract

y+LAT1 (encoded by *SLC7A7*), together with y+LAT2 (encoded by *SLC7A6*), is the alternative light subunits composing the heterodimeric transport system y+L for cationic and neutral amino acids. SLC7A7 mutations cause lysinuric protein intolerance (LPI), an inherited multisystem disease characterized by low plasma levels of arginine and lysine, protein‐rich food intolerance, failure to thrive, hepatosplenomegaly, osteoporosis, lung involvement, kidney failure, haematologic and immunological disorders. The reason for the heterogeneity of LPI symptoms is thus far only poorly understood. Here, we aimed to quantitatively compare the expression of *SLC7A7* and *SLC7A6* among different human cell types and evaluate y+LAT1 and y+LAT2 contribution to arginine transport. We demonstrate that system y+L‐mediated arginine transport is mainly accounted for by y+LAT1 in monocyte‐derived macrophages (MDM) and y+LAT2 in fibroblasts. The kinetic analysis of arginine transport indicates that y+LAT1 and y+LAT2 share a comparable affinity for the substrate. Differences have been highlighted in the expression of *SLC7A6* and *SLC7A7* mRNA among different cell models: while *SLC7A6* is almost equally expressed, *SLC7A7* is particularly abundant in MDM, intestinal Caco‐2 cells and human renal proximal tubular epithelial cells (HRPTEpC). The characterization of arginine uptake demonstrates that system y+L is operative in renal cells and in Caco‐2 where, at the basolateral side, it mediates arginine efflux in exchange with leucine plus sodium. These findings explain the defective absorption/reabsorption of arginine in LPI. Moreover, y+LAT1 is the prevailing transporter in MDM sustaining a pivotal role in the pathogenesis of immunological complications associated with the disease.

## 1 INTRODUCTION

Lysinuric protein intolerance (LPI; MIM 222700) is a recessively inherited autosomal disease caused by mutations of *SLC7A7* (solute carrier family 7A member 7; MIM #603593), coding for y+LAT1 protein, that is part of the cationic amino acid (CAA; arginine, lysine and ornithine) transport system y^+^L.^1^, ^2^ Patients manifest with low plasma levels of arginine and lysine and typically display protein‐rich food intolerance and secondary urea cycle disorders; other symptoms are heterogeneous, and include failure to thrive, recurrent vomiting, hepatosplenomegaly, osteoporosis, lung involvement, kidney failure, haematologic abnormalities and immunological disorders.^3^

y+LAT1, together with y+LAT2 (encoded by *SLC7A6*), is one of the alternative light subunits composing the heterodimeric transport system y^+^L; the heavy chain is, instead, the glycoprotein 4F2hc (encoded by *SLC3A2*), that is required for the proper expression of the transporter on the plasma membrane.^4^ This transport system selectively couples the sodium‐independent efflux of CAA to the influx of neutral amino acids (leucine, glutamine) and sodium, working as an antiport.^5^ The activity of system y^+^L has been initially described in erythrocytes, then it has been identified in placenta, platelets, skin fibroblasts, hepatocytes, small intestine and kidney; in polarized epithelia, it is mainly located onto the basolateral cell membrane, where it mediates the transport of cationic amino acids from the renal and intestinal epithelia towards the bloodstream.^6^ Previous studies performed by our group have shown that monocytes/macrophages express high levels of *SLC7A7* and, consistently, that arginine transport by system y^+^L is definitely impaired in mononuclear cells isolated from LPI patients.^7^ On the contrary, other LPI cells, such as fibroblasts and erythrocytes,^8^, ^9^ do not display any defect in arginine transport, suggesting that the alternative transporter y+LAT2 can compensate the defect of y+LAT1 in these models.

In light of these findings and of clinical evidence,^10^ the most accredited pathogenetic hypothesis for LPI symptoms is that circulating cells as monocytes/macrophages, that actually manifest the transport defect, are responsible for the multiorgan complications of the disease and, in particular, for pulmonary and immunological ones. However, since little is known about the relative contribution of SLC7A7/y+LAT1 and SLC7A6/y+LAT2 to arginine transport in other tissues, in this study, we aimed to compare their actual expression and activity among different cell types, paying particular interest to intestinal and renal cells, targets of LPI disease.

## 2 MATERIALS AND METHODS

### 2.1 Cell cultures

Human monocytes were isolated from buffy coats of normal, healthy donors, provided by the Unit of Immunohematology and Transfusion of the Azienda Ospedaliero‐Universitaria of Parma (local ethics committee approval # 43899, 03/12/2015), as previously described.^11^ Monocyte‐derived macrophages (MDM) were obtained by incubating monocytes for 6 d in RPMI1640 supplemented with 10% endotoxin‐free foetal bovine serum (FBS) and 50 ng/mL of recombinant human GM‐CSF. Normal human fibroblasts, obtained from a 15‐year‐old healthy donor,^12^ were cultured in high glucose Dulbecco's modified Eagle's medium (DMEM) with 4 mmol/L glutamine. Human renal proximal tubular epithelial cells (HRPTEpC), purchased from Sigma‐Aldrich (Italy), were grown in REGM medium, as indicated by the manufacturer; only cells between first and third passages were used. Caco‐2 intestinal epithelial cells were obtained from American Type Culture Collection (ATCC) and routinely grown in DMEM. Polarized Caco‐2 monolayers were established by seeding cells at a density of 3 × 10^5^/mL on polyester (PET) membrane Transwell^®^ inserts (12 mm diameter, 0.4 µm pore size, Falcon) in 24‐well plates. About 200 μL and 700 µL culture media were added to the apical and basolateral compartments of the insert, respectively. Cell cultures were employed 7 days after seeding when monolayers exhibited high transepithelial electrical resistance (TEER values > 600 Ohm·cm^2^ using an epithelial voltmeter (EVOM, World Precision Instruments)). The integrity of cell monolayers was preserved after the experiments. EpiIntestinal™, a model of human small intestinal tissue (SMI‐100), was supplied by MatTek Corporation (Ashland) and cultured with medium provided by the manufacturer. Alveolar epithelial A549 and Calu‐3 cells, deriving from a human lung adenocarcinoma, were obtained from ATCC and cultured in high glucose DMEM and in Eagle's Minimum Essential Medium (EMEM), respectively. K562, a chronic myeloid leukaemia cell line, was obtained from Deutsche Sammlung von Mikroorganismen und Zellkulturen GmbH and maintained as suspension cultures in RPMI1640 medium. Lymphoblasts, kindly provided by Dr G. Sebastio (Department of Paediatrics of the University Federico II of Naples; Italy), were grown in suspension in RPMI1640 medium. Human CD3^+^ lymphocytes were a kind gift of Dr Laura Passerini (San Raffaele Telethon Institute for Gene Therapy, Milan, Italy). EpiAirway**™** system, a 3‐D human‐derived tracheal/bronchial epithelial cells (AIR‐200‐PE6.5), was supplied by MatTek Corporation and cultured as indicated by the manufacturer. Unless otherwise specified, the growth medium for all these cell types was supplemented with 10% foetal https://www.sciencedirect.com/topics/biochemistry-genetics-and-molecular-biology/bovine serum (FBS) and 1% penicillin/streptomycin; all cells were routinely cultured under physiological conditions (37.5°C, 5% CO_2_, 95% humidity).

### 2.2 Gene silencing

Gene silencing was performed employing HiPerFect Transfection Reagent, AllStar Negative Control (scrambled siRNA, Cat.# SI03650318) and FlexiTube short interfering RNAs (SLC7A6/y+LAT2 siRNA, Cat.# SI00058681 and SLC7A7/y+LAT1 siRNA, Cat.# SI00058660) by Qiagen®. Fibroblasts were transfected according to the protocol provided by the manufacturer. For MDM, isolated monocytes (3 × 10^6^/mL) were maintained in antibiotic‐free RPMI1640 medium containing 50 ng/mL GM‐CSF for 72 hours, then washed with PBS and incubated in serum‐free medium containing 60 µL/mL HiPerFect Transfection Reagent and 2 nmol/L (final concentration) scrambled or specific siRNA; after 6 hours, 0.3 volumes of complete growth medium were added. Cells were employed after 96 hours.

### 2.3 RT‐qPCR analysis

Total RNA was isolated with GeneJET RNA Purification Kit and reverse transcribed with RevertAid First Strand cDNA Synthesis Kit (Thermo Fisher Scientific); the amount of the genes of interest (SLC7A6/y+LAT2 and SLC7A7/y+LAT1) was, then, determined using TaqMan® Gene Expression Assays by Thermo Fisher Scientific (Cat# Hs00187757_m1, Hs00909952_m1, and Hs03855120_g1, respectively), and expressed as numbers of mRNA molecules upon normalization to that of the housekeeping gene (RPL15, Ribosomal Protein Like 15).^13^

### 2.4 L‐Arginine uptake

**Figure 1:**
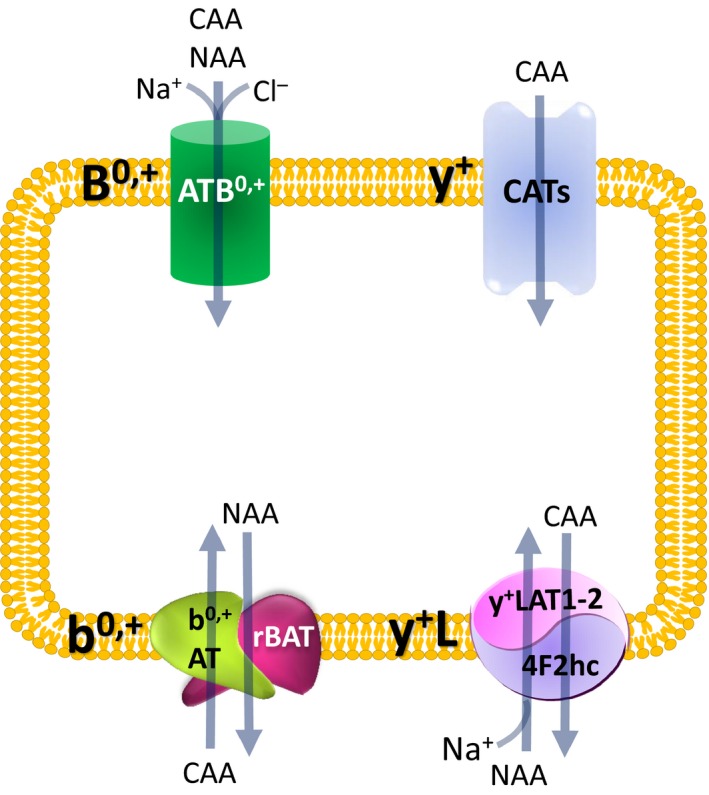
Schematic representation of transport systems for cationic amino acids in a hypothetical cell. CAA, cationic amino acids; NAA, neutral amino acids

In mammalian cells, four distinct transport mechanisms, namely systems y^+^, y^+^L, b^0,+^ and B^0,+^, mediate cationic amino acid uptake.^5^ System y^+^ operates a sodium‐independent and membrane potential–dependent unidirectional transport specific for cationic amino acids, while the other three transporters accept both cationic and neutral amino acids.^14^ ATB^0,+^ is a highly concentrative system, being energized by the transmembrane gradients of Na^+^ and Cl^−^, as well as by membrane potential. System b^0,+^ is Na^+^‐independent and mediates the obligatory exchange of cationic amino acids and cystine with neutral amino acids with 1:1 stoichiometry. ATB^0,+^ and b^0,+^are expressed in the apical membrane of epithelial cells. Lastly, the ubiquitary expressed system y^+^L transports cationic amino acids in the absence of sodium and neutral amino acids in the presence of the cation. Under physiological conditions, it operates as an efflux route by exporting cationic amino acids from inside the cells in exchange with neutral amino acids plus sodium; this system is localized at the basolateral side of epithelial cells. The features of these transport systems are resumed in Figure 1.

For uptake studies, cells, cultured onto 96‐well trays, were washed twice at 37°C with Earle's Balanced Salt Solution (EBSS, composition in mmol/L: 117 NaCl, 1.8 CaCl_2_, 5.3 KCl, 0.9 NaH_2_PO_4_, 0.8 MgSO_4_, 5.5 glucose, 26 Tris/HCl, pH 7.4), and incubated for 1 minute in the same solution containing [^3^H]arginine (50 µmol/L, 5 μCi/mL) in the absence or presence of leucine (2 mmol/L) or leucine plus lysine (2 mmol/L). For the measurements of arginine uptake in the absence of sodium, a modified EBSS (NMG‐EBSS) was employed in which NaCl and NaH_2_PO_4_ were replaced by N‐methyl‐D‐glucamine and choline salts, respectively. Uptake was terminated by the removal of uptake solution followed by three rapid washes with cold 300 mmol/L urea. Cell monolayers were extracted in ethanol and radioactivity counted in a Wallac Microbeta Trilux2 liquid scintillation spectrometer (Perkin Elmer). L‐arginine uptake was normalized for protein content, determined directly in each well by using a modified Lowry method.^15^ Uptake is expressed as nmol/mg of protein/min. The activity of system b^0,+^ was calculated as the difference between arginine transport in the absence of inhibitors (total) and the transport measured in the presence of leucine in the absence of sodium. System y^+^L was calculated as the difference between arginine transport in the absence of inhibitors (total) and the transport measured in the presence of leucine in the presence of sodium, after the subtraction of b^0,+^ quote, when present. System y^+^ activity was determined as the quote of transport further inhibited by lysine in the presence of sodium.

The apparent kinetic parameters *K*_m_ (Michaelis constant) and *V*_max_ (maximum transport rate) of arginine uptake were calculated by non‐linear regression fitting according to the Michaelis‐Menten equations:

(1) $$v=\frac{V_{\max}\times \left[S\right]}{K_{m}+\left[S\right]}$$

for a single saturable component, where *v* is the initial influx, *V*_max_ is the maximal influx and *K*_m_ is the Michaelis constant.

### 2.5 Determination of arginine transcellular fluxes in polarized Caco‐2 cells

Caco‐2 cell monolayers, grown in Transwell^®^ (24‐well plates) to confluence, were washed with Earle's Balanced Salt Solution (EBSS) at 37°C and incubated, at the apical side, with 100 µL EBSS containing [^3^H]arginine (50 µmol/L, 10 µCi/mL); the basolateral compartment was incubated in 550 µL EBSS, in the absence or in the presence of 10 mmol/L leucine. At the times indicated, 100 µL of the solution in the basolateral compartment was collected and replaced with an equal volume of fresh EBSS solution. Radioactivity in each sample was counted in a Wallac Microbeta Trilux2 liquid scintillation spectrometer (Perkin Elmer).

### 2.6 Statistics

Prism^®^ 5.0 GraphPad software has been employed for the statistical analysis; statistical significance was calculated with Student's *t* test for unpaired data, unless stated otherwise. Differences were considered significant when *P* < .05.

### 2.7 Materials

Endotoxin‐free foetal bovine serum (FBS) was purchased from EuroClone (Italy); L‐[2,3,4‐3H]Arginine monohydrochloride (43 Ci/mmol) was purchased from Perkin‐Elmer (Italy) and GM‐CSF from Vinci‐Biochem (Italy). Unless otherwise stated, Sigma‐Aldrich (Italy) was the source of all the other chemicals.

## 3 RESULTS

### 3.1 Biochemical features of y+LAT1 and y+LAT2 transporters

In a previous contribution, we showed that the activity of system y^+^L was impaired in LPI monocytes and alveolar macrophages, but readily detectable in fibroblast‐like mesenchymal cells obtained from an LPI patient, as well as in fibroblasts from healthy donors.^7^ Based on the analysis of gene expression, we there suggested that a normal expression of *SLC7A6*/y+LAT2 could compensate the mutation of *SLC7A7*/y+LAT1 in LPI fibroblasts conferring them normal arginine uptake, whereas the predominant expression of y+LAT1 in monocytes and macrophages could explain the defective transport observed in LPI cells.

**Figure 2:**
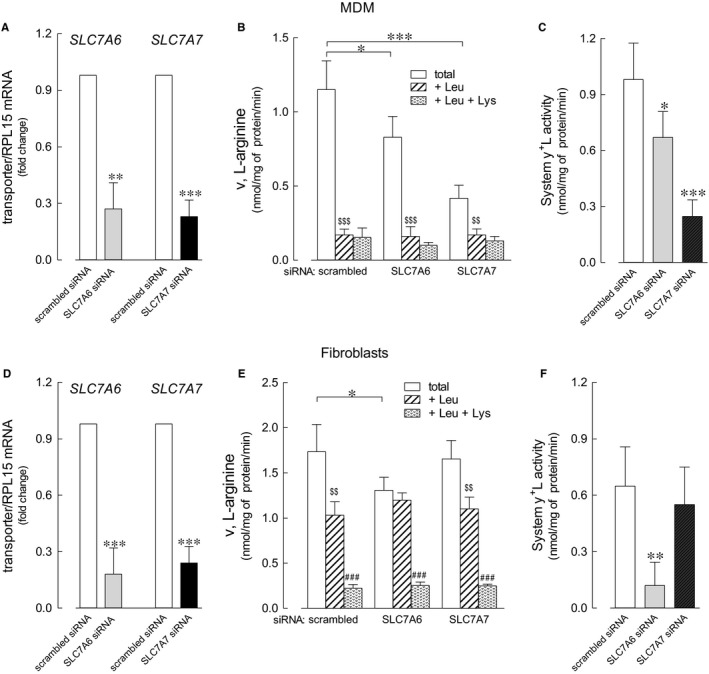
Effect of SLC7A6 and SLC7A7 silencing on arginine transport in monocyte‐derived macrophages (MDM) and fibroblasts. MDM and fibroblasts were transfected with scrambled or with *SLC7A6* or *SLC7A7* siRNA, as described in Methods. Panels A and D: the expression of both *SLC7A6* and *SLC7A7* in silenced cells was referred to that of scrambled siRNA (=1). Bars are mean ± SEM of three independent experiments, each performed in duplicate. **P < .01, ***P < .001 vs scrambled siRNA calculated with one sample t test. Panels B and E: arginine transport was measured through a 1‐min incubation in EBSS containing [^3^H]arginine (0.05 mmol/L; 2 μCi/mL) in the absence or presence of 2 mmol/L leucine (+ Leu) or 2 mmol/L leucine + 2 mmol/L lysine (+Leu +Lys) (see Methods). Data are mean ± SD of three independent determinations in a representative experiment, that, repeated twice, gave comparable results. *P < .05, ***P < .001 vs scrambled siRNA; ^$$^ *P* < .01, ^$$$^ *P* < .001 vs total uptake; ^###^ *P* < .001 vs +Leu. Panels C and F: data shown in panels B and E were employed to calculate the relative contribution of system y^+^L as the difference between total transport and transport measured in the presence of leucine. * *P* < .05, ** *P* < .01, *** *P* < .001 vs scrambled siRNA

In order to verify this hypothesis, we here first tried to discriminate the relative contribution of the two transporters in fibroblasts and monocytes‐derived macrophages (MDM), by selectively silencing one or the other gene. Data obtained (Figure 2) indicate that the expression of both transporters was efficiently reduced by the specific siRNA, both in MDM (panel A) and in fibroblasts (panel D). In MDM, *SLC7A6* gene silencing resulted in a slight reduction of total arginine transport (panel B), whereas a more marked inhibition (more than 65%) was observed in *SLC7A7*‐silenced cells. Since the presence of leucine almost completely suppressed arginine uptake under any condition, we can conclude that in MDM, the activity of system y^+^L, calculated as the quote of arginine uptake inhibited by leucine (panel C), is only marginally hindered by *SLC7A6* siRNA, while almost completely abolished upon *SLC7A7* silencing. Conversely, in fibroblasts only the down‐regulation of *SLC7A6* proved effective in reducing arginine transport, while *SLC7A7* silencing did not (panel E); moreover, since leucine exerted its inhibitory effect in *SLC7A7*‐, but not in *SLC7A6*‐silenced cells (panel F), we can conclude that system y^+^L‐mediated arginine transport in these cells is likely accounted for by y+LAT2 protein.

**Figure 3:**
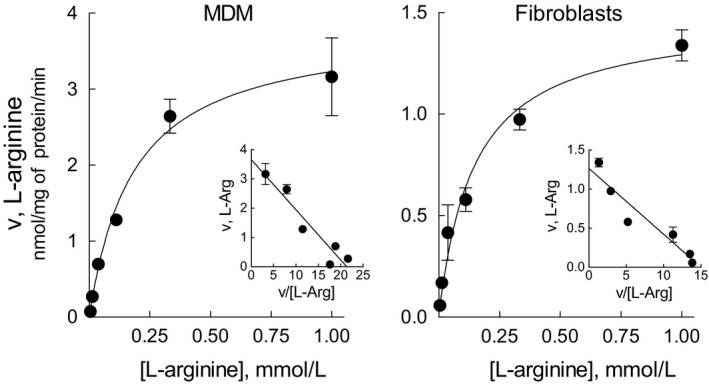
Kinetic constants of arginine transport through y+LAT1 in MDM, and through y+LAT2 in fibroblasts. MDM and fibroblasts were transfected for 72 h with SLC7A6 and SLC7A7 siRNA, respectively. After gene silencing, cells were incubated for 1 min in the presence of the indicated concentrations (from 0.04 to 1 mmol/L) of [^3^H]arginine (from 2 to 10 μCi/mL), in the absence and in the presence of 5 mmol/L leucine. Non‐linear fitting of the data, obtained by calculating y^+^L activity as the difference between total uptake and the uptake obtained in the presence of leucine, was performed employing Equation (1) (see Methods). Inserts in each graph show the Eadie‐Hofstee transformations of uptake. Straight lines are drawn employing the values of the kinetic parameters given by non‐linear regression. Points are mean ± SD of three independent determinations in a representative experiment, repeated twice with comparable results

Starting from these findings, we next addressed the biochemical features of the transporters by performing the kinetic analysis of arginine transport in silenced cells; more precisely, we measured the kinetic constants of system y^+^L (calculated as the difference between total transport and the transport measured in the present of leucine) in MDM silenced for *SLC7A6* (hence measuring the activity of the sole y+LAT1) and in fibroblasts silenced for *SLC7A7* (hence measuring the activity of the sole y+LAT2). Results, presented in Figure 3, indicate that the Michaelis constants (*K*_m_) of the two alternative proteins are very similar (0.182 ± 0.035 mmol/L and 0.145 ± 0.028 mmol/L for MDM and fibroblasts, respectively) and, thus, that they have a comparable affinity for arginine. V_max_ values are, instead, different (3.822 ± 0.24 and 1.479 ± 0.09 nmol/mg of protein/min for MDM and fibroblasts, respectively), suggesting differences in the number of functional transporters on the membrane or in their intrinsic activity due to in‐trans substrate availability.

### 3.2 Tissue‐specific pattern of expression of SLC7A6/y+LAT2 and SLC7A7/y+LAT1

**Figure 4:**
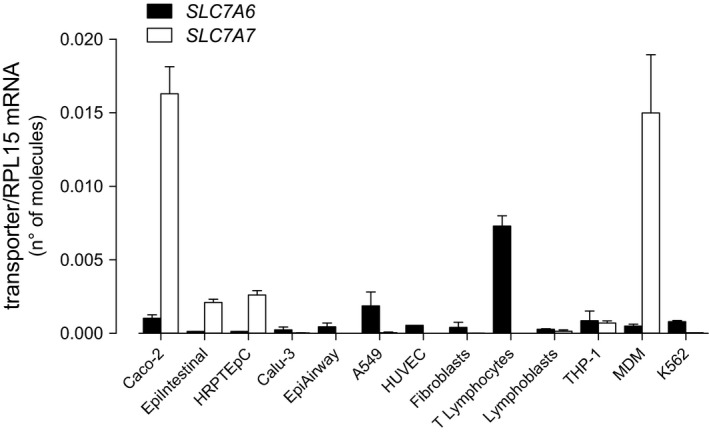
Expression of SLC7A6 and SLC7A7 in different human cells. After RNA extraction and reverse transcription, cDNA was employed as template for qPCR *SLC7A6* and *SLC7A7* mRNA levels are expressed as number of molecules after normalization for that of *RPL15* gene (see Methods). Bars are mean ± SEM of three independent determinations obtained in different set of cells, each performed in duplicate

Next, we addressed the differential expression of *SLC7A6*/y+LAT2 and *SLC7A7*/y+LAT1 in different tissues through a quantitative RT‐qPCR analysis of the two genes in various cell models. As shown in Figure 4, *SLC7A7* was impressively expressed in MDM and intestinal Caco‐2 cells, and relatively high in human renal proximal tubular (HRPTEpC) and in small intestine epithelial cells (EpiIntestinal™); with the exception of THP‐1 monocytic cell line, the gene was undetectable in all other models. On the contrary, the expression of *SLC7A6* was almost comparable in all cells tested, with CD3^+^ T lymphocytes showing the highest mRNA levels.

### 3.3 Arginine transport in renal and intestinal epithelial cells

**Figure 5:**
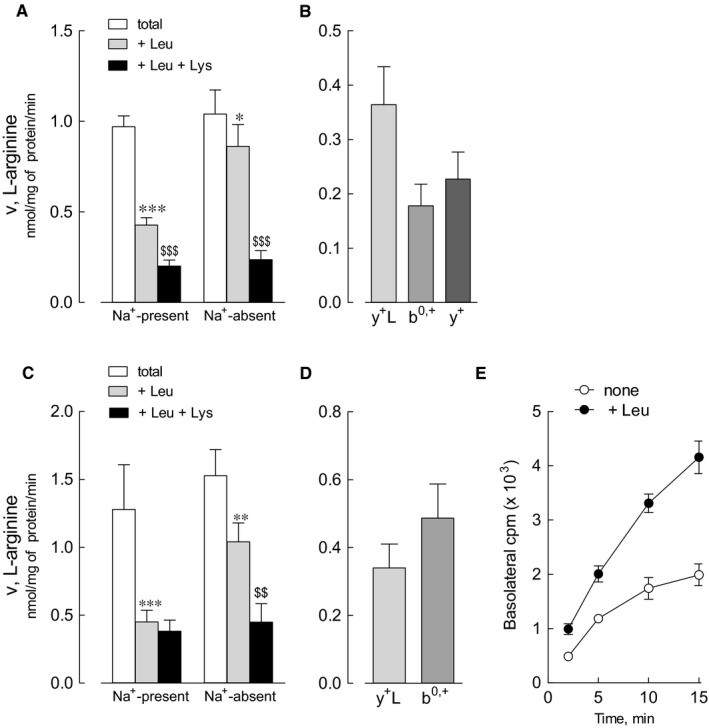
Arginine transport in human renal proximal tubular epithelial cells (HRPTEpC) and in Caco‐2 cells. Panels A and C. HRPTEpC (panel A) and Caco‐2 (panel C) cells, grown in 96‐well plates, were washed in EBSS in the presence or absence of sodium, and arginine transport was measured through a 30 s incubation in the same solution containing [^3^H]arginine (0.05 mmol/L; 5 μCi/mL) in the absence or presence of 2 mmol/L leucine (+ Leu) or 2 mmol/L leucine+2 mmol/L lysine (+ Leu +Lys) (see Methods). Bars are mean ± SEM of three independent experiments each performed in quadruplicate. * *P* < .05, ** *P* < .01, *** *P* < .001 vs total uptake; ^$$^ *P* < .01, ^$$$^ *P* < .001 vs +Leu. Panels B and D. Data shown in Panels A and C were employed to calculate the relative contribution of systems y^+^L, b^0,+^ and y^+^ (see Methods). Panel E. For the measurement of arginine transcellular flux in polarized Caco‐2 monolayers, cells, cultured onto inserts, were incubated at the apical side in 100 µL EBSS containing L‐[^3^H] arginine (0.05 mmol/L; 8 μCi/mL) and at the basolateral side in 550 µL EBSS in the absence or presence of 10 mmol/L leucine (+ Leu). At the times indicated, aliquots of 100 µL of the basolateral medium were collected, replaced with fresh medium and counted for radioactivity. Points are mean ± SD of three independent determinations within a representative experiment that, repeated twice, gave comparable results

Data of gene expression are consistent with the notion that beside immune cells, intestine and kidney are the main targets of LPI defect; however, little is thus far known about system y^+^L activity in human renal and intestinal cells. Figure 5 shows the characterization of arginine transport in primary renal proximal tubular epithelial cells (HRPTEpC) and in Caco‐2 cells, the representative in vitro model of the intestinal barrier. The relative contribution of each transporter has been assessed through a methodology previously used in other cell models.^16^ Arginine uptake was comparable in the absence and in the presence of sodium in both renal (panel A) and intestinal (panel C) cells, thus excluding a significant contribution of sodium‐dependent transport systems, such as B^0,+^. In the absence of sodium, leucine significantly inhibited arginine uptake, demonstrating that system b^0,+^ is operative in these cells. Moreover, since the inhibitory effect of leucine was much more evident in the presence of the cation, we can conclude that also system y^+^L, typically inhibited by neutral amino acids only in the presence of sodium, is functional. Under this experimental condition, the addition of 2 mmol/L lysine to leucine further lowered arginine transport in HRPTEpC, indicating the operation of system y^+^ in these cells; on the contrary, the lack of inhibition by lysine in Caco‐2 cells indicates that the contribution of system y^+^ in this model is at most marginal. In summary, arginine uptake in renal cells is mediated by the activity of systems y^+^L, b^0,+^ and y^+^ (panel B), while only systems y^+^L and b^0,+^are operative in the intestinal model (panel D).

In these latter, the transcellular flux of arginine has been also monitored in polarized monolayers; to this end, cells grown on inserts were incubated, at the apical side, in the presence of labelled arginine and, at the basolateral side, in EBSS, either in the absence or presence of leucine. As demonstrated in panel E, the efflux of arginine through the basal side of the monolayer was trans‐stimulated by the presence of leucine, indicating that, in the intestinal barrier, system y^+^L likely cooperates to the absorption of the amino acid by exchanging intracellular arginine with extracellular leucine plus sodium.

## 4 DISCUSSION

In this study, we address the relative contribution of y+LAT1 and y+LAT2 to the uptake of cationic amino acids in different human cell models, so as to help to shed light onto the organ specificity of LPI manifestations. By interacting with the heavy chain 4F2hc, these two proteins form alternative heterodimeric transporters belonging to system y^+^L, that is responsible for the exchange of dibasic amino acids with neutral amino acids and Na^+^.

On a molecular basis, y+LAT1 and y+LAT2 share an high grade of sequence homology 6; accordingly, we show here that their affinity for the common substrate arginine is very similar (0.182 mmol/L for y+LAT1 and 0.145 mmol/L for y+LAT2). Differences in V_max_ values are likely consistent with the diverse expression of the transporters in the two cells models. To the same extent, differences in the expression of *SLC7A6* and *SLC7A7* mRNAs among various in vitro cell models suggest a different contribution of y+LAT2 and y+LAT1 transporters to arginine uptake in the different districts. In particular, while *SLC7A6* appears quite homogeneously expressed, with CD3^+^ lymphocytes showing the highest expression, *SLC7A7* is readily detectable only in monocyte‐derived macrophages and in intestinal and renal cells, where y+LAT1 is hence supposed to operate most of system y+L‐mediated arginine transport.

These findings indicate that the relative abundance of the expression of *SLC7A7* in the different cell types accounts for LPI signs: the presence of *SLC7A6*/y+LAT2 is expected to compensate the defective activity of y+LAT1 transporter in most tissues, while the almost exclusive presence of the mutated *SLC7A7*/y+LAT1 in others would be the reason for the onset of LPI complications. Hence, our finding of an high expression of *SLC7A7* in macrophages, intestinal and renal cells against very low levels of *SLC7A6* is consistent with the role of these models as the preferential targets of the disease and can help explaining the pathogenesis of several symptoms of LPI.

As reported by Broer, both renal and intestinal LPI phenotypes are explained by the presence of the sole system y+L transporter in the basolateral membranes of these tissues 17; indeed, the lack of a functional y+LAT1 consequent to *SLC7A7* mutations is realistically the reason for the impaired absorption‐reabsorption of cationic amino acids, and, in turn, for the low plasma levels of arginine and other cationic amino acids in LPI patients. Our study of arginine transcellular fluxes in polarized layers of Caco‐2 cells confirms that system y^+^L in this model is operative at the basolateral side, blowing out arginine in exchange with leucine plus sodium. We also demonstrate here for the first time that arginine transport in primary human renal epithelial cells is mediated by system y^+^L, along with systems b^0,+^ and y^+^. Unexpectedly, in both models of epithelial cells we did not find any activity of the sodium‐dependent system B^0,+^, the high‐affinity carrier for neutral and cationic amino acids. Literature evidence indicates that this transporter, codified by ATB0,+/*SLC6A14*, localizes in murine colon, lung and eye,^18^ while, in humans its expression is detected at low levels in normal cells, but significantly induced under pathological conditions, such as tumours and inflammation.^19^ In our hands, system B^0,+^ is not operative in Caco‐2 human intestinal cells and in primary renal cells, a finding congruent with lack of expression of *SLC6A14* in these models, as well as in MatTek's 3D tissue model of human small intestine (EpiIntestinal) (data not shown). Consistently, Ahmadi et al recently reported the absence of expression of this gene in Caco‐2 cells.^20^ In contrast, system B^0,+^ is readily detectable in respiratory epithelium where, beside arginine, it accepts carnitine.^13^, ^16^

As far as the immune system is concerned, experimental and clinical evidence sustains a key role for circulating cells in the onset of LPI pulmonary and immunological complications.^21^ To this concern, we demonstrated in years that system y^+^L‐mediated arginine transport is severely compromised in LPI monocytes 7 and that phagocytosis is defective in monocytes‐derived macrophages (MDM) obtained from LPI patients,^22^ suggesting a role for these cells in LPI‐associated immune dysfunctions. Now, we demonstrate here that, although expressing the mRNA for both *SLC7A6* and *SLC7A7*, MDM largely depend on y+LAT1 for arginine transport. Gene silencing of both transporters causes, indeed, a significant decrease of system y+L activity; however, it is only *SLC7A7* silencing that almost completely suppresses system y+L‐mediated arginine transport, while the knockdown of *SLC7A6*/y+LAT2 only modestly impact on the uptake of the amino acid, confirming that y+LAT1 is the predominant transporter in monocytes/macrophages.

It is important to remind, however, that the mechanisms linking the genetic defect in innate immune cells to the clinical manifestations of the disease still remain to be elucidated. The original hypothesis was that the impairment of arginine efflux due to y+LAT1 defect in LPI immune cells leads to an intracellular accumulation of arginine responsible for an increased production of the inflammatory mediator nitric oxide.^23^ More recently, however, we demonstrated that the silencing of *SLC7A7* gene in THP‐1 monocytes associates with the induction of an inflammatory phenotype, no matter arginine intracellular concentration,^24^ thus proposing to enrol LPI in the group of auto‐inflammatory diseases. This finding is consistent with the occurrence in LPI patients of hemophagocytic lymphohistiocytosis (HLH), a disorder caused by an uncontrolled and self‐sustained cytokine‐driven immune activation of T lymphocytes and macrophages.^25^ We show here, in accordance with data by Werner et al,^26^ that CD3^+^ lymphocytes almost exclusively express *SLC7A6*; as a consequence, no alteration of arginine transport is expected to occur in LPI T lymphocytes. According to this finding, the pathogenetic mechanism for LPI‐associated HLH is expected to involve pathological monocyte/macrophages, rather than T cells.

## CONFLICT OF INTEREST

The authors have no conflict of interest to declare.

## AUTHORS' CONTRIBUTIONS

BMR and AB designed the experimental plan. FF and RV performed in vitro experiments; BMR, AB and VD analysed the results and wrote the paper. All authors read and approved the final manuscript.

## Data Availability

Research data are not shared.
